# Morbidity of Inguinofemoral Lymphadenectomy in Vulval Cancer

**DOI:** 10.1100/2012/341253

**Published:** 2012-01-04

**Authors:** A. A. Soliman, M. Heubner, R. Kimmig, P. Wimberger

**Affiliations:** ^1^Department of Obstetrics and Gynaecology, Shatby Maternity University Hospital, University of Alexandria, Port Said Street, El Shatby, Alexandria 21526, Egypt; ^2^Department of Gynaecology and Obstetrics, University of Duisburg-Essen, Hufelandstraße 55, 45122 Essen, Germany

## Abstract

*Background*. The aim of this study is to detect possible risk factors for development of short- and long-term local complications after inguinofemoral lymphadenectomy for vulval cancer. *Methods*. This retrospective cohort study included 34 vulval cancer patients that received inguinofemoral lymphadenectomy. The detected complications were wound cellulitis, wound seroma formation, wound breakdown, wound infection, and limb lymphoedema. Followup of the patient ran up to 84 months after surgery. *Results*. Within a total of 64 inguinofemoral lymphadenectomies, 24% of the inguinal wounds were affected with cellulitis, 13% developed a seroma, 10% suffered wound breakdown, 5% showed lower limb edema within a month of the operation, and 21.4% showed lower limb edema during the long-term followup. No significant correlation could be found between saphenous vein ligation and the development of any of the local complications. The 3-year survival rate in our cohort was 89.3%. *Conclusions*. Local complications after inguino-femoral lymphadenectomy are still very high, with no single pre-, intra-, or postoperative factor that could be incriminated. Saphenous vein sparing provided no significant difference in decreasing the rate of local complications. More trials should be done to study the sentinel lymph node detection technique.

## 1. Introduction

Vulval cancer is the fourth most common gynecologic malignancy accounting for 5% to 8% of all female genital tract malignancies [1]. The incidence of vulval cancer is on the rise and may be due to the increased life expectancy of the females in the developed societies [2]. Since the en bloc dissection through a butterfly incision that was advocated by Taussig in the 1940s [3], many modifications were adopted in order to decrease the associated high morbidity after such radical surgical procedure. Modifying the surgical technique through using the triple groin incision [4] instead of the original en bloc surgical resection and using saphenous vein sparing technique [5] succeeded partially in decreasing the high local morbidity associated with surgery for inguinofemoral nodal clearance. Wound cellulitis occurs in 25–39% of patients, wound breakdown in 17–31% of patients, and lymphedema in 28–39% of patients [6–8]. Despite the decrease in the incidence of morbidities after inguinofemoral lymph node clearance, the rate is still high. Inguinofemoral lymph node clearance can be omitted in vulval lesions smaller than 2 cm with infiltration not deeper than 1 mm [1]. Contralateral lymphadenectomy can only be omitted in small T1 unilateral lesions when the ipsilateral lymph nodes are free, but it is standard if the lesion is 1 cm or less close to the midline [9]. The following study aims at analyzing possible factors associated with development of postoperative wound complications.

## 2. Patients and Methods

This retrospective cohort study was performed in the Department of Gynaecology and Obstetrics, University of Duisburg-Essen, Germany. Patients' records were analysed in the period between January 2002 to December 2009 looking for patients with the diagnosis of invasive Vulval Cancer that received a radical vulvectomy with inguinofemoral lymphadenectomy. Microinvasive vulval cancer patients, patients with relapse of vulval cancer, and patients with advanced inguinal disease with palpable, fixed, or ulcerated inguinal lymph nodes were excluded from our analysis. Inguinofemoral lymphadenectomy is performed through a separate skin incision between the anterior superior iliac spine and the pubic tubercle, parallel to the inguinal ligament and with a length of about 7 cm. All the lymphofatty tissue extending from 2 cm above the inguinal ligament to the sartorius muscle laterally and the medial border of the adductor longus medially are removed. These tissues are removed along with the cribriform fascia and the lymphatic tissues running along the femoral vessels and in between them from just below the inguinal ligament till about 2 cm proximal to the beginning of Hunter's canal when the femoral vessels are crossed by the Sartorius muscle. In our institution, the greater saphenous vein is spared whenever possible. Closed suction drainage was universally used in all the operated cases. Lymph drainage massage, compression stockings, and subcutaneous antithrombosis prophylaxis are standard treatments applied to all the patients. The following characteristics were registered: patient's age, cardiovascular diseases, diabetes mellitus, amount of intraoperative bleeding, saphenous vein ligation, number of days of suction drainage, the average daily and the total amount of fluid accumulated in the drains, TNM staging, number of lymph nodes resected as well as the number of infiltrated lymph nodes, development of wound cellulitis (local redness and swelling of the wound edges with no evidence of infection), wound seroma (accumulation of fluid in the wound space without opening the wound edges that can be detected sonographically), wound breakdown (defined as opening of one-third or more of the wound length that requires secondary sutures), wound infection (documented with bacteriological smear), and development of limb lymphedema. The sites of relapse and the time to relapse from the date of the operation are registered during the followup. Long-term complications are also registered during the follow-up period. The registered data was tabulated and analysed using Chi square and Fischer exact tests for categorical data. *P* values of less than 0.05 are considered statistically significant.

## 3. Results

Analyzing the operative records in the aforementioned time period yielded 76 vulval cancer patients with only 34 patients that received radical inguinofemoral lymphadenectomy, 30 of them had bilateral inguinofemoral lymphadenectomy, and 4 patients had unilateral inguinofemoral lymphadenectomy summing up into a total of 64 inguinal lymphadenectomy procedures. The median age of our patients was 56.5 years (range from 23 to 86 years). 31% of our cohort aged between 45 and 55, and 53% were 55 years of age or older. Age was independent of the development of inguinal wound complications. 37.5% (*n* = 12) of the cohort had heart diseases: hypertension, coronary artery diseases, arrhythmia, or valvular diseases. 20% of the patients (*n* = 7) had diabetes mellitus. 73% (*n* = 47) of the operated groins had 7 or more lymph nodes excised per groin, 44.5% (*n* = 28) had 10 or more lymph nodes excised; these numbers reflect the radicality of the adopted surgical procedure. 31% of the patients were stage T1b (TNM classification) (*n* = 12), 65% were T2 (*n* = 21) and only one patient had T3. The frequency of development of inguinal wound complications was 24.2% (*n* = 15) wound cellulitis, 9.7% (*n* = 6) wound breakdown, 12.5% (*n* = 8) wound seroma, and 3.2% (*n* = 2) wound infection. 4.8% (*n* = 3) developed early limb lymphoedema while on long-term followup 21.4% (*n* = 6) showed lower limb lymphoedema. Many pre-, intra-, and post-operative factors were investigated. Cardiac diseases and the amount of intraoperative bleeding were both significantly related to the development of wound cellulitis (*P* = 0.022 and *P* = 0.026, resp.). Cardiac diseases were also significantly related to the development of wound breakdown (*P* = 0.001). The number of infiltrated lymph nodes was significantly related to the development of wound seroma (*P* = 0.005). Saphenous vein ligation carried no significant correlation to the development of any of the studied wound complications. The duration of wound suction drainage, the amount of exudate accumulated in the drains, and the number of excised lymph nodes per groin were not significantly correlated to the development of any of the studied complications. Only one vulval wound was complicated with infection, and another vulval wound was complicated with wound dehiscence.  Table 1 shows the frequency of simultaneous occurrence of wound complications in the same groin in our cohort. A significant correlation was found between the development of wound cellulitis and the subsequent development of wound breakdown (*P* < 0.001).

The patients were followed up after surgery for a median period of 30 months (range from 6 to 84 months). 6 patients were lost to follow up. 3 deaths due to cancer were registered, and they were all pT2. Time interval to death due to cancer ranged from 5 to 30 months with a median of 10.5 months. 1 Mortality was due to complications of cancer treatment, while the other two were due to cancer recurrence. The 3-year survival rate is 89.3%. Local recurrence was registered in 17.8% (*n* = 5) of cases. The median time to recurrence was 11 months (range from 6 to 38 months). No inguinal recurrences were reported. Development of early wound complications did not correlate to the development of long-term limb-lymphoedema.

## 4. Discussion

Since the introduction of the radical vulvectomy and bilateral inguinofemoral lymphadenectomy through the butterfly skin incision by Taussig [3] in the 1940s, many modifications were introduced to the operative technique aiming at decreasing the reported high complications rate without compromising the radicality of the procedure or affecting the survival and recurrence rates that is why the triple incision procedure was introduced to replace the en bloc surgical excision [4, 10]. Another modification was introduced when Zhang et al. published their retrospective analysis comparing saphenous vein sparing to saphenous vein ligation during the inguinal lymphadenectomy and its impact on the development of complications. They found significant decrease in the development of short-term lower limb phlebitis in the saphenous vein spared group; otherwise, no significant differences were noted between the two groups as regards the development of post-operative wound seroma, acute inguinal wound cellulitis, or lymphocyst formation [11]. However, we proved in our study that saphenous vein ligation did not significantly affect the development of any of the studied postoperative complications including limb lymphedema formation, even on long-term followup. While Gould et al. found a significant correlation between the development of early wound complications and the development of late complications like lymphedema [6], Gaarenstroom and his group failed to find any correlation between early and late complications in their published cohort [7]. In our study, development of inguinal wound cellulitis was significantly correlated to the subsequent development of early wound breakdown. However, the incidence of wound complications or early lymphedema was not correlated to the development of leg lymphedema or any other local complications in the long-term followup. The relatively lower complications rates registered in our cohort in comparison to other publications (see Table 2) might be explained but cannot be confirmed, by the routine use of compression stockings, daily wound cleansing, prophylactic antibiotic intake, and daily lymph drainage massage.

The observation that the presence cardiac diseases was significantly correlated with the development of wound cellulitis, and the subsequent development of wound breakdown could be explained but not validated by the possible limited tissue perfusion in the presence of cardiovascular disease with consequent affection of the healing power of the tissues. However, during our literature review, we could not recognize any other author who mentioned this observation studied or discussed it; thus, this point will remain open for further research to validate it and possibly explain its mechanism. The observation that the amount of intraoperative bleeding was significantly correlated with the development of wound cellulitis was also not studied or mentioned by other authors and remains open for further validation and explanation. In our opinion, excessive intraoperative bleeding invited more electrocoagulation of the tissues or inserting hemostatic sutures, which will increase tissue ischemia and hence healing power postoperatively, but still this remains open for further discussion and validation. 

In spite of the aforementioned modifications to the surgical technique, the incidence of wound complications was still high. This invited trying other additional materials and techniques to decrease the high morbidity. One Gynecologic Oncology Group study was designed to examine the efficacy of a commercially available FDA-approved fibrin sealant-applied to the inguinal wound compared to conventional wound closure methods. However, the 137 patients included in this study did not show any significant difference in the rate of development of leg lymphedema in any subgroup. The inguinal wound complications rates did not significantly differ between both treatment modalities, and the development of vulval wound infection was even significantly more frequent in the fibrin sealant treatment arm [12]. The success of the sentinel lymph node detection technique in the treatment of breast cancer and melanomas made it possible to study its results in vulval cancer patients. Since the first report of sentinel lymph node detection techniques in vulval cancer by Levenback et al. using isosulfan blue dye [13], many authors have tried to prove the efficacy and accuracy of such a conservative operative technique in dealing with early stage cases. Almost all the reports focused on the detection rates of affected lymph nodes in comparison with the current radical surgical methods, and they scrutinized the false negative rates and methods to decrease them to an acceptable minimum, but very few reports to date addressed the effect of such a technique in decreasing the high rate of operative morbidity after standard radical inguinofemoral lymphadenectomy [14–19]. Most of the authors report a very low false negative detection rate ranging between 0% and 9%, but Radziszewski et al. reported a low negative predictive value of the sentinel lymph node procedure of 92% concluding that it is not possible to recommend it as a standard procedure for treating early vulval cancer [19]. Van Der Zee et al. in the largest published prospective trial to date studying the sentinel lymph node detection in early vulval cancer reported an 11.7%, 4.5%, and 1.9% incidence of inguinal wound breakdown, inguinal wound cellulitis, and leg lymphoedema, respectively, in the sentinel lymph node only group. This was the biggest trial to analyze the short- and long-term complications of sentinel lymph node-detection technique. It showed a 97% 3-year survival with 2.3% groin recurrence rate and 12.3% local recurrence rate for patients with negative sentinel lymph nodes [20]. It is, therefore, clear from the above data that there is no single risk factor that affects the development of local inguinal complications. It is also obvious that saphenous nerve sparing techniques do not necessarily decrease the incidence of local complications. Sentinel lymph node technique is proved successful in decreasing the local complications rate, but its sensitivity and specificity are still needed to be further examined by means of well-designed randomized controlled trials.

## Figures and Tables

**Table 1:** Frequency (*n*) of simultaneous complications in the same groin.

	Wound cellulitis	Wound breakdown	Wound seroma formation	Wound infection	Early leg lymphedema
Wound cellulitis		5 (*P* < 0.001)	3	1	1
Wound breakdown	5 (*P* < 0.001)		1	0	0
Wound seroma formation	3	1		0	0
Wound infection	1	0	0		0
Early leg lymphedema	1	0	0	0	

**Table 2:** Comparison between the incidence of wound complications in the current cohort and other published data.

	Current study	Gaarenstroom et al. [7]	Gould et al. [6]
Wound cellulitis	24.2% (*n* = 15)	Not studied	35.4%
Wound breakdown	9.7% (*n* = 6)	11% (*n* = 21)	19.4%
Wound seroma	12.5% (*n* = 8)	27% (*n* = 50)	13.1%
Wound infection	3.2% (*n* = 2)	27% (*n* = 51)	Not studied
Early lower limb lymphedema formation	4.8% (*n* = 3)	21% (*n* = 40)	4.8%
